# An Image Stabilization Optical System Using Deformable Freeform Mirrors

**DOI:** 10.3390/s150101736

**Published:** 2015-01-15

**Authors:** Qun Hao, Xuemin Cheng, Jiqiang Kang, Yuhua Jiang

**Affiliations:** 1 Beijing Key Lab. for Precision Optoelectronic Measurement Instrument and Technology, School of Optoelectronics, Beijing Institute of Technology, Beijing 100081, China; E-Mail: flyinskyxm@hotmail.com; 2 Graduate School at Shenzhen, Tsinghua University, Shenzhen 518055, China; E-Mails: cheng-xm@mail.tsinghua.edu.cn (X.C.); jqkang@hku.hk (J.K.)

**Keywords:** geometric optics, computation methods, stabilization system

## Abstract

An image stabilization optical system using deformable freeform mirrors is proposed that enables the ray sets to couple dynamically in the object and image space. It aims to correct image blurring and degradation when there is relative movement between the imaging optical axis and the object. In this method, Fermat's principle and matrix methods are used to describe the optical path of the entire optical system with a shift object plane and a fixed corresponding image plane in the carrier coordinate system. A constant optical path length is determined for each ray set, so the correspondence between the object and the shift free image point is used to calculate the solution to the points on the surface profile of the deformable mirrors (DMs). Off-axis three-mirror anastigmats are used to demonstrate the benefits of optical image stabilization with one- and two-deformable mirrors.

## 1. Introduction

Imaging systems integrated into aircraft or land-based vehicles vibrate during vehicular translation and rotation motion. Thus, to capture sharp images of still target objects, image stabilization techniques are required. In a stabilized system each point on the still object plane, before and during movement, corresponds to a single fixed image point. This is achieved by sensing the angular positions about its center of rotation with an inertial sensor when the host platform or a pointing mirror in the system undergoes disturbances, or by calculating the image sequence interframe offset with image processing techniques [1–4]. Then it is stabilized by generating the necessary counter-rotations with the spin and torquer motors [5–12] which necessitates larger moment of inertia resulting in a large system and a slow response rate. Thus a combined servo mechanism and stabilization frame are generally suggested in the systems to achieve a stabilization accuracy of 0.10 mrad without increase of the volume [13–15], e.g., the coarse/fine combination two-level stabilization technique in helicopters, the two-axis line-of-sight stabilization system mounted on a stabilized platform for vehicle navigation, and *etc*.

Many researchers have considered the use of deformable mirrors (DMs) in adaptive optics [16–21], beam shaping [22,23] and more recently, zoom lens designs [24–26]. These techniques take advantage of the ability of DMs to correct aberrations and change focus dynamically [27,28], resulting in higher image quality without the need for moving components. The application of deformable mirrors could potentially improve current imaging methods significantly.

In our previous work [24], a zoom lens employing deformable mirrors as the focal-length-variable elements has been discussed. In this paper, the application of DMs to compensate the residual vibration from vibration isolation platform using its deformable freeform surface profiles is investigated. It is expected that the abilities of zooming and stabilizing can be integrated in the cameras by the use of the same DMs. The use of the ray tracing technique in the design of the surface profile is also discussed. First, the optical principles of the image stabilization method are described. Second, the ray tracing paths from the object and image points are established from their point coordinates and normal vectors, using matrix methods and Fermat's principle. The initial curves of the DM profile can be then calculated, ensuring the image shift compensation and aberration corrections are realized from these parameters. Finally, a set of initial curves in three-dimensional space are combined and described, using a polynomial to determine the initial freeform surface profile for the image stabilized optical systems.

A stabilized system is dynamically achieved when the surface profile of the DM is controlled using a set of micro actuators. The field of view of the system remains an optical image stabilization method. In the imaging path, if the deformable surface is used instead of a mirror plate, there is more scope to control the ray tracing paths for image shift compensation and aberration balance in the vibration process. It can also respond quickly, accurately change the surface shape with a deviation of less than 0.2 μm, and has real-time control, which is useful for use in mobile devices, aerial vehicles, and high resolution camera modules [29]. It can also improve the mechanical and electrical characteristics of these instruments. The characteristics of asymmetry present a challenge to the design of freeform mirrors in imaging systems, as the image shift must be corrected without affecting image quality. Optimization algorithms in general optical design software usually start from a set of predefined parameters and a predetermined configuration. Therefore, a proper initial design, with a parameterized description of the DM profiles using the proposed method, would improve the resulting solution/convergence, or further optimize the design of the image stabilization system.

## 2. Methods

### 2.1. Image Shift Compensation Using DMs

**Figure 1.**
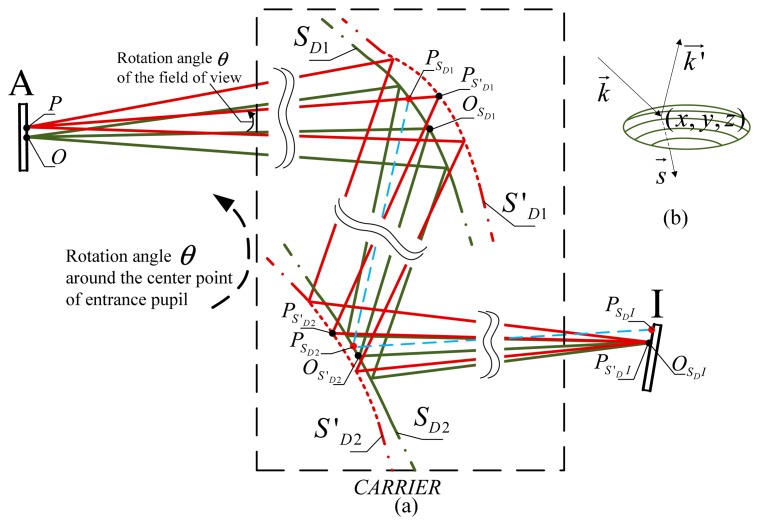
(**a**) Illustrates the ray tracing paths for image shift compensation using DMs. Double wavy lines in the optical system indicate optical elements on either side of the DMs. Green solid lines show the segments on the original profiles of DM surfaces *S*_*D*_1__ and *S*_*D*_2__ placed between the object plane and the image plane. Red dotted lines show the segments on the changed profiles of DM surfaces *S′*_*D*_1__ and *S′*_*D*_2__ for image shift compensation. Green solid lines trace rays from point *O*, the initial location, through the optical system towards point *O_S_D_I_*. Points *O*_*S*_*D*1__ and *O*_*S*_*D*2__ are the intersections of the incident rays and the reflected rays on the DMs. The blue dashed lines from points *P*_*S*_*D*1__, *P*_*S*_*D*2__ and *P_S_D_I_* represent ray tracing from the shift point location *p* in the initial optical path and locate a shift point *P_S_D_I_* on the image plane. Red solid lines are the rays from point *P*, the shift point location on the object plane, tracing the compensated optical path towards point *P_S′_D_I_* using the changed profiles of DM surfaces; (**b**) Illustrates the relationship between the vectors of the reflected rays and the normal line on one point of the mirror surface in space.

In this section, we describe the process of image shift correction using DMs. The carrier is translated and rotated in the global coordinate system; however, for the rays originating from the same object point, it is appropriate to simplify to a rotation angle of *θ* to the optical axis, as the variation of the object distance is small when the object is at infinity. Therefore the coordinates of the same object point are shifted during the relative motion between the object and the carrier in the carrier coordinate system, shown in Figure 1a. The optical system is established by the use of the tilted DMs and other optical refractive or reflective elements. Point *O* forms the initial point location on the object plane A. Point *O_S_D_I_* is the corresponding image point on image plane *I* when using the original surfaces profiles *S*_*D*_1__ and *S*_*D*_2__.

A point *P_S_D_I_* on the image plane *I* is formed when the object point is shifted from point *O* to point *P*. We calculate the coordinates (*x*, *y*, *z*) and normal vectors *s⃗* of points *P_S′_D_I_* and *P*_*S*′_*D*2__ on new profiles of the DM surfaces *S′*_*D*_1__ and *S′*_*D*_2__, off which the ray is reflected, to correct the image shift on the image plane when point *P_S_D_I_* is corrected to point *P_S_D_′I_*. Thereafter, by using surfaces *S′*_*D*_1__ and *S*′_*D*_2__, the image points remain fixed. The optical path differences are at the same time constrained so the image quality of the optical system does not deteriorate. Figure 1b illustrates the relationship between the vectors of the incident ray and the reflected ray *k⃗*, $\vec{k\prime }$, and the normal vector *s⃗*.

### 2.2. Approximation Method for DM Design

As stated, the DM method was applied to the design of an image stabilization system, OIS DM. For practical consideration, one- and two-DMs in the optical system will be discussed. In this section, we describe the initial parameters for OIS DM design. These were calculated dynamically by applying a constant optical path length for each ray set, which is the same defined value as that of the optical path of the initial ray sets, calculated by choosing one point on each surface profile along the ray paths. The OIS DM surfaces are regarded as piecewise segments that map the tilted ray sets for fixed image points. The profiles of the surfaces in the optical system were calculated to find out which integrability conditions needed to be satisfied [30,31], with continuity between adjacent segments on the surface, which can interpolate with a higher order polynomial freeform surface for further optimization.

**Figure 2.**
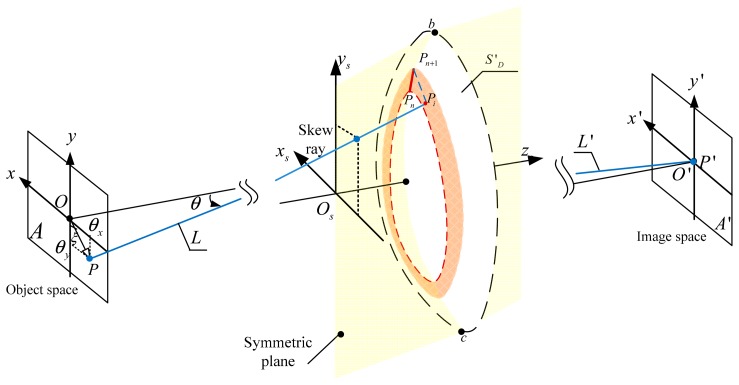
OIS DM segment design through the use of ray tracing in 3D space.

The OIS DM system is a tilted optical system that has the characteristic of plane symmetry, shown in Figure 2. This illustrates the rotation *θ* of the field of view for one ray in 3D object space, as the location of the object point is shifted from point *O* to point *P*. The angle between $\bar{OP}$ and the *y* axis of the object plane is *ξ* and the object distance is *L*. It is assumed that the OIS DM optical system maintains the characteristic of plane symmetry in the process of image stabilization. The locations of the points on the image plane are fixed, while the values of the field of view for the corresponding points in the object space are changed by *θ_y_* and *θ_x_* along the *y* and *x* directions, when *θ_y_* = arctan(cos*ξ* tan*θ*) and *θ_x_* = arctan(sin*ξ* tan*θ*). Ray tracing through the optical system established the coordinates and normal vector of the intersection points on the DM surfaces for the arbitrary skew rays in the full field of view. First, the ray bundle from the points on the symmetric plane was traced from the object space and the image space. Forward ray tracing from point (*θ_x_*, *θ_y_*) in the object space and backward ray tracing from point (0, 0) in the image plane determined the center points of the OIS DM. We determined consecutive points sequentially in the symmetric plane for the OIS DMs using the ray tracing technique calculated from point *P_n_*, in which *P_n_* and *P_n_*_+_*_1_* are a pair of consecutive points and $\bar{P_{n}P_{n+1}}$ is one segment on the OIS DM surface *S′_D_*, shown in Figure 2. The profile of surface *S′_D_* was divided into adjacent circular zones using the calculated points (*P*_0_, ⋯ *P_n_*, *P_n_*_+1_⋯) on the OIS DM. Second, the intersection point on the OIS DMs out of the symmetric plane was calculated by the use of skew ray tracing [32]. Using the circular zones defined by points *P_n_* and *P_n_*_+_*_1_*, the intersection *P_i_* of the skew ray with the OIS DM was constrained and optimized to find the optimal optical path length. Third, segments between points *P_n_*, *P_n_*_+_*_1_* and points *P_n_*_+_*_1_*, *P_i_* were interpolated to satisfy the integrability condition for splines of degree 3, with continuity between adjacent segments. This ensured that the interpolated points and the normal points matched for further optimization. Finally, the intersection points with the OIS DM were calculated from the interior portion to the edge of the surface sequentially.

**Figure 3.**
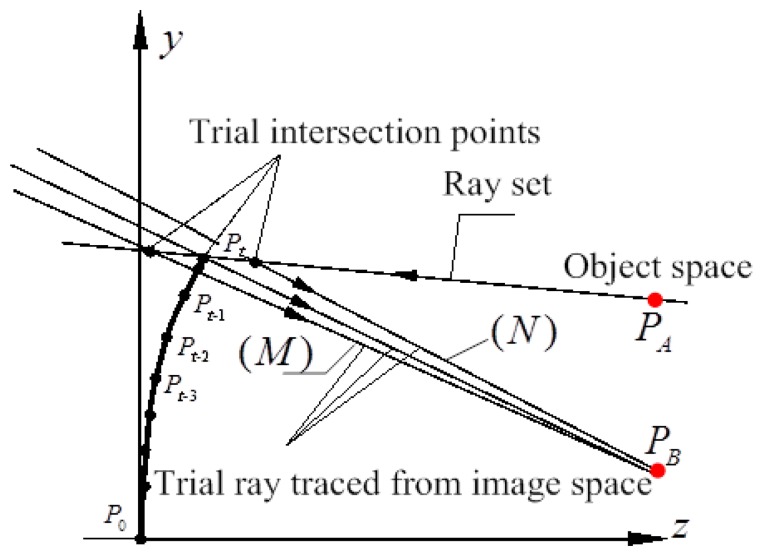
Schematic illustration of surface profile calculation for DMs.

The intersection point on the OIS DM surface profile of the incident ray and the reflected ray can be described through its coordinates and vectors. According to Snell's principle, real ray tracing for any ray sets can be a complex problem in the design process of the initial configuration. The parameters were therefore deduced from the paraxial ray tracing of the incident and the reflected rays, and calculated using matrix methods. Thus the point coordinate and the slope of the ray set were calculated using the radius of curvature at the intersection point and the corresponding distances, shown in Figure 3. *P_A_*(*x_A_*, *y_A_*, *z_A_*) and *P_B_*(*x_B_*, *y_B_*, *z_B_*) are points on the incident ray $\bar{P_{A}P_{t}}$ and the reflected ray $\bar{P_{t}P_{B}}$ rays before and after DM, respectively. *P_t_*(*x_t_*, *y_t_*, *z_t_*) is the intersection point on the OIS DM surface. The distance between *P_A_* and *P_t_* is *L_1_*. The distance between *P_B_* and *P_t_* is *L_2_*. Thus the reflected ray $\bar{P_{t}P_{B}}$ can be calculated by Equations (1) and (2) [33]:

(1) $$\left(\begin{array}{c} x_{B}\\ \alpha_{B} \end{array}\right)=\left(\begin{array}{cc} 1 & L_{2}\\ 0 & 1 \end{array}\right)\times \left(\begin{array}{cc} 1 & 0\\ -2/R_{x} & 1 \end{array}\right)\times \left(\begin{array}{cc} 1 & L_{1}\\ 0 & 1 \end{array}\right)\times \left(\begin{array}{c} x_{A}\\ \alpha_{A} \end{array}\right)$$

(2) $$\left(\begin{array}{c} y_{B}\\ \beta_{B} \end{array}\right)=\left(\begin{array}{cc} 1 & L_{2}\\ 0 & 1 \end{array}\right)\times \left(\begin{array}{cc} 1 & 0\\ -2/R_{y} & 1 \end{array}\right)\times \left(\begin{array}{cc} 1 & L_{1}\\ 0 & 1 \end{array}\right)\times \left(\begin{array}{c} y_{A}\\ \beta_{A} \end{array}\right)$$

where *α_A_* and *α_B_* are the slopes of the ray at points *P_A_* and *P_B_* in the *x* direction. *R_x_* is the corresponding radius of curvature at the intersection point. *β_A_* and *β_B_* are the slopes of the ray at points *P_A_* and *P_B_* in the *y* direction and *R_y_* is the corresponding radius of curvature at the intersection point.

For two intersecting rays $\bar{P_{A}P_{t}}$ and $\bar{P_{t}P_{B}}$, the approximate point was calculated in the neighbor of intersection point *P_t_* where the optical path difference Ω was constrained and optimized to find the optimal optical path length, so at each surface the optical path length was computed for the ray, satisfying Fermat's principle. The Newton-Raphson method is applied iteratively at each point. For each ray traced in the optical space of DM, trial rays were traced backward from image point. As a first approximation, we chose the intersection of the trial ray with a certain vector (*L*) from the fixed imaging point *O_S_D_I_* in image space. Trial rays of vector (*M*) and (*N*), shown in Figure 3, were used to compute a good approximation to the derivative *f′* in Equation (3):

(3) $$f^{\prime }\approx \frac{\Omega^{\left(M\right)}-\Omega^{\left(N\right)}}{\gamma^{\left(M\right)}-\gamma^{\left(N\right)}}$$

where Ω was a function *f*(*γ*), Ω^(^*^M^*^)^ and Ω^(^*^N^*^)^ were the optical path difference for trial rays (*M*) and (*N*), *γ*^(^*^L^*^)^, *γ*^(^*^M^*^)^ = (1 + Δ*γ*)·*γ*^(^*^L^*^)^ and *γ*^(^*^N^*^)^ = (1 − Δ*γ*)·*γ*^(^*^L^*^)^ were the slope of trial rays (*L*), (*M*) and (*N*) in the local y-z plane, Δ*γ* was a small increment. The intersection point was determined when a sufficiently small residual ΔΩ was achieved.

The ray path was then checked to compute the value of the final image shift, or the distance between point *P_S′_D_I_* and point *O_S_D_I_*, by tracing along the tilted ray and reflection off the intersection points, towards the point *P_S′_D_I_* on the image plane *I*. The intersection point will be maintained when the image shift is constrained within a certain range, e.g., an accuracy of 0.1 pixels. As a consequence of intersection points being calculated and interpolated using a spline, the infinitesimal profile gaps are limiting, and the design process results in the DM surface profiles *S*′*_D_*_1_ and *S*′*_D_*_2_.

## 3. Results and Discussion

**Figure 4.**
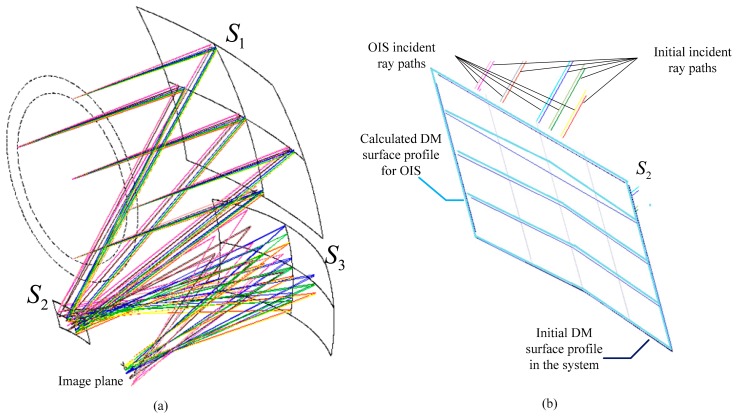
(**a**) Ray tracing paths overlay the configurations for the initial system and the one DM OIS system with the calculated surface profile; and (**b**) an enlarged drawing showing the initial DM surface profile in the system as dark blue segments, and the calculated DM surface profile for OIS as the light blue segments, indicating the initial incident and the OIS incident ray paths.

In this section, the design results of the image stabilization system using DMs are investigated. First, an off-axis three-mirror anastigmat, shown in Figure 4, is used to study the effect of OIS DMs controlling image shift. With only reflective surfaces in the system, the relationship is between the image stabilization values and the deflection variation of the deformable mirrors in the optical system. An image stabilization system was also simulated using a commercial DM from OKO Technologies [34]. The intersection points on the OIS DM calculated above were interpolated with a 10th order polynomial freeform surface with plane symmetric characteristics, and the profile was then integrated into the optical design software. The assumption is that the image sensor array consists of 1024 × 768 pixels. The experimental results of the proposed method and the electrical stabilization method are then demonstrated and compared.

### 3.1. OIS DM Using an Off-Axis Three-Mirror Anastigmat

An off-axis three-mirror anastigmat, shown in Figure 4, is an optical system of three tilted mirrors *S*_1_, *S*_2_ and *S*_3_. The initial parameters of the system were as follows: wavelength of 500 nm, focal length of 250 mm, f number of 2.5, field of view in *x* direction of ±2.5° and in *y* direction of ±0.5°. The system achieved an accuracy of 0.1 pixels or 0.002 mm.

We discussed the design results for optical image stabilization where one mirror and two mirrors in the system were replaced by the DM, respectively. A rotation angle was also introduced in the system carrier so the values of the field of view in the object space were varied by *θ*. As the corresponding image points inside an image area were fixed in the same way as the initial configuration, the coordinates and normal vectors of the points on the surface profile of the DMs were calculated using the analytic function and the approximation method, presented in Section 2.2. The image quality of the OIS DM system was then analyzed in CODE V [35] with the calculated surface profiles.

The system with one DM OIS at surface *S*_2_ is referred to as a single DM OIS system. When two OIS DMs are present at surfaces *S*_2_ and *S*_3_, this is referred to as a two DMs OIS system. Calculations were therefore carried out for both systems to evaluate the effects of image shift compensation and image balance. The *θ* variation was selected in the range of 0.1° when the image quality could be kept at the same level as the initial anastigmat. Considering the characteristics of plane symmetry, the variations of field of view were selected to be located on the edge of the rotation angle along seven directions, which is proper for image analysis, and are listed in Table 1.

**Figure 5.**
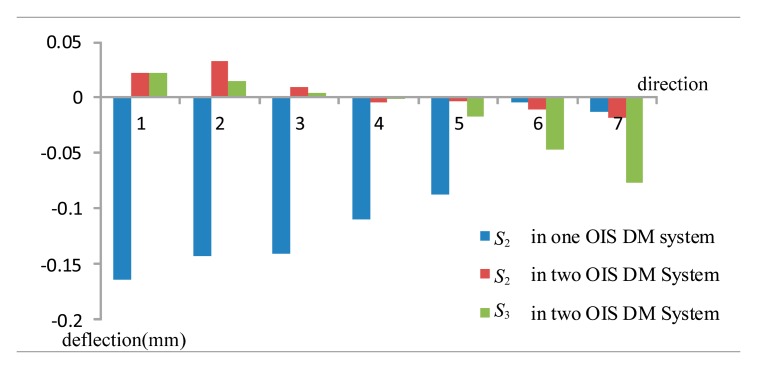
Deflections of the surface at the DM center point for the variations along seven directions, with different colors for the corresponding DMs.

We calculated the DM surface profiles for the one DM OIS system using variations of field of view along seven directions in Table 1. Figure 4a shows the design results when taking the variation No. 1 in Table 1, with ray tracing paths in the overlay configurations for the anastigmat of the initial surface profile and the one DM OIS system using the calculated surface profile. The enlarged drawing, Figure 4b, illustrates the segments on the initial DM surface profile and those on the calculated DM surface profile for OIS. The values of the image shift for the corresponding ray sets are smaller than 0.0019 mm as the image stabilization accuracy of 0.1 pixels for the system is 0.002 mm. The deflections of the surface *S*_2_ at the DM center point for the variations along seven directions are calculated according to the coordinates and the normal vector of the points, as listed in Figure 5.

The design of the two DMs OIS system was also investigated. DM surface profiles at surfaces *S*_2_ and *S*_3_ were calculated along the seven directions for the variation of the field of view by 0.1°. Next, the surface profile was integrated in the system. The image shifts for corresponding ray sets were evaluated and the values smaller than 0.0018 mm, constrained in the image stabilization accuracy of 0.1 pixels. The deflections of the surface *S*_2_ at the DM center point for the variations along all directions are indicated in Figure 5.

The values of the deflection for surface *S*_2_ in the single DM OIS system, also shown in Figure 5, decreased as the variation changed from No. 1 to No. 7. The sign of the deflection for surfaces *S*_2_ and *S*_3_ changed with the rotation directions in the two DMs OIS system. However, larger variation of the field of view would require larger deflection of the DM surface to achieve the same image stabilization accuracy. The deflections of the surface in the two DMs OIS system were smaller than those in the one DM system as the rotation angle takes the directions 1, 2, 3, 4 and 5. This was due to the determined surface profiles of the initial anastigmat, as surface *S*_2_ is tilted and occupies one portion of the integral surface with the surface vertex located outside of the ray path. To satisfy the requirements of Fermat's principle and the image stabilization, a larger deflection of the surface is required, as the rotation of the field of the view (positive direction) incurs a larger incident ray angle on surface *S*_2_.

### 3.2. OIS DM System Using a Commercial DM

This section describes the result of an optical system using the presented DM technique. The focal length of the system was 146 mm and the full field of view was ±1.75°. The variation of the field of view for the system was 0.017°, or 1′, which is acquired from the motion sensors. For an image sensor array of 1027 × 768 pixels, the value of 0.1 pixel accuracy is 0.0006 mm in the system.

**Figure 6.**
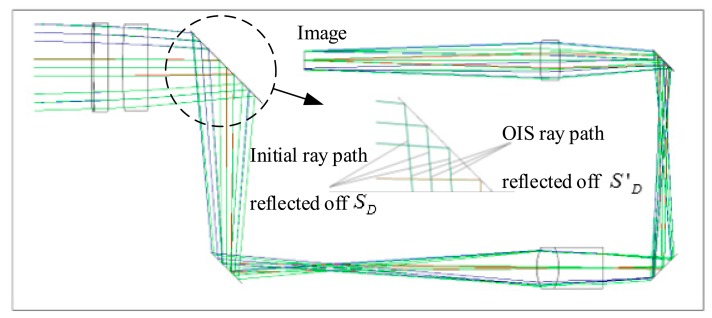
The overlayed drawing of the configurations for the initial ray paths using surface *S_D_* and the OIS ray paths using surface *S*′*_D_*.

**Figure 7.**
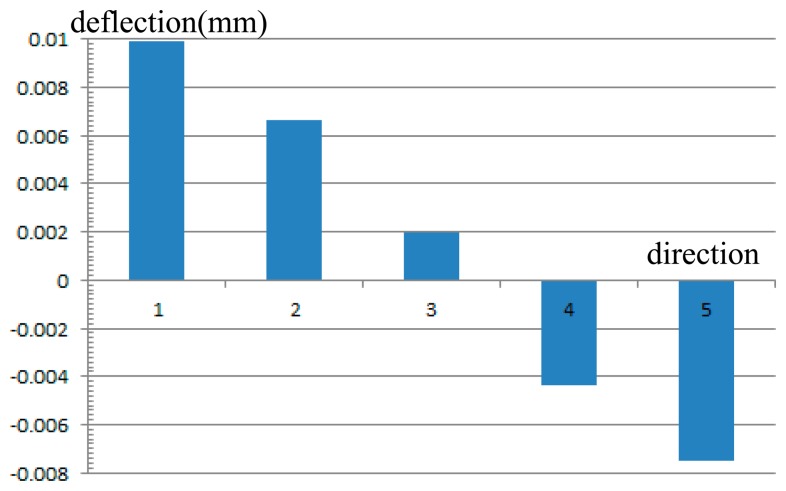
Maximum deflections of the DM surface as the field of view is varied along five directions.

DM surface profiles for the OIS system were also calculated along the five directions listed in Table 2 for a field of view variation of 0.017°. With an image stabilization accuracy of 0.0006 mm, the design result of the proposed OIS system using one DM is shown in Figure 6. For the initial ray paths using surface *S_D_* and the OIS ray paths using surface *S′_D_*, the two configurations are overlayed and described using the image plane as reference. The enlarged drawing indicates the ray paths incident on and reflected off surfaces *S_D_* and *S′_D_*. The diameter of the DM was 20 mm. The deflection of the surface was optimized and constrained to 0.010 mm, as indicated in Figure 7. Constrained image aberration is readily available with commercial DMs from OKO Technologies.

The results in Section 3.1 showed dynamic image shift compensation in the constraints of 0.002 mm for 0.1° of rotation in the single mirror case and in the dual mirror case. A smaller deflection of the surfaces was observed in the dual mirror case. In this section, the compensation simulation using the commercial DM was constrained to within 0.0006 mm for one minute of rotation. Compared to other methods currently being used in the field, these results are optimal with an image stabilization accuracy of 0.1 pixels.

**Figure 8.**
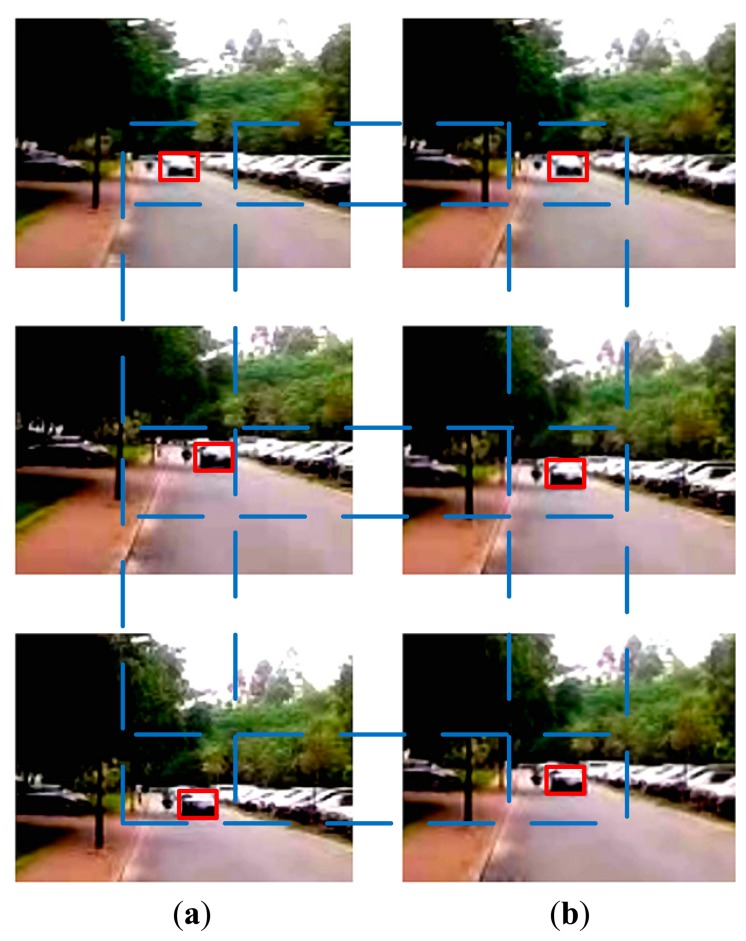
(**a**) the vibrating images; (**b**) those compensated by the optical stabilization method, using two DMs, the car in the image sequences was marked in red line.

**Figure 9.**
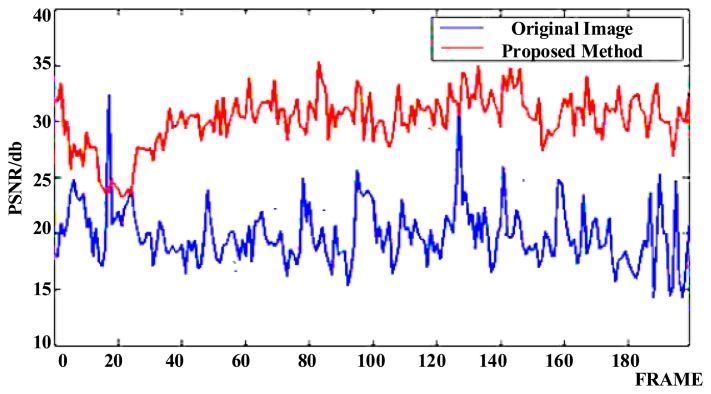
Peak Signal to Noise Ratios (PSNR) for two experiments: Those for the original image and the proposed method, using two DMs.

The images shown in Figure 8a are three frames of captured video from the CCD in the experimental platform with two commercial DMs (one DM was 20 mm and another DM was 10 mm in diameter in the optical path, with 37 actuators controlling the surface shape) for image stabilization, showing the effects of vibration, as the vehicle is driven and a car approaches from the opposite direction. The optical stabilization method using two DMs was then applied and the resulting images were also captured at the same frame. The vehicle and the car were controlled to proceed at the same speed in both experiments. The image shifts are shown to be compensated in Figure 8b, where the features of the car are more easily distinguished. The values of Peak Signal to Noise Ratios (PSNR) are used to estimate the image quality and are shown in Figure 9. This illustrates that the proposed method produces better quality images.

## 4. Conclusions

In this study, we propose a novel image stabilization technique, utilizing Fermat's principle and matrix methods to determine the location of intersection points dynamically on the surface of a deformable mirror, by describing the image shift for the entire optical system. This enabled the calculation of the intersection points on the surface profile of the deformable mirror using a series of coupled ray sets between the object and image planes. Using these equations, the ray path was then traced forward along the tilted ray and off the intersection points, towards the image point. The value of the final image shift was then computed. The intersection points were maintained when the image shift was constrained within a certain range. These intersection points were interpolated with a 10th order polynomial freeform surface of plane symmetric characteristic, so the profile can be integrated and optimized in the optical design software. This method was used to simulate the control of image shifts for one- and two-mirror anastigmats and for a commercial DM. Ray tracing results, which calculated the mirror profiles in an image stabilization system for an off-axis three-mirror anastigmat, demonstrated that the image shift could be constrained to within 0.002 mm using one DM when the system was rotated by 0.1°. With two DMs and the same rotation, the shift is within 0.002 mm, with a much smaller deflection of the surfaces. A commercial DM was also implemented and controlled the image shift to within 0.0006 mm when the system was rotated by 1 min. Evaluation of experimental results for the proposed method demonstrated that the image quality improved.

The high response time of the DMs, and their ability to correct aberrations, demonstrate their potential to improve high-end cameras for vehicle navigation, thermal imaging, small satellite remote sensing, *etc*. Compared to other methods currently being used in the field, these results are optimal with an image stabilization accuracy of 0.1 pixels. The results of this study have the potential to improve image stabilization, and by continuing to investigate the optimization technique of the initial configuration, the ray tracing algorithm, through calculating the intersection point and the profile calculation method for the plane symmetric freeform surface.

## Figures and Tables

**Table 1.** Variations of the field of view for the system along seven directions. Variations No. 1 and 7 are selected along the y directions, No. 5 along the x direction, No. 2 and 6 along the diagonal directions, and No. 3 and 5 are selected according to the aspect factor by the field of view of the system in y and x directions.

***No.***	**1**	**2**	**3**	**4**	**5**	**6**	**7**
*θ_x_*(°)	0.000	0.071	0.098	0.100	0.098	0.071	0.000
*θ_y_*(°)	0.100	0.071	0.020	0.000	−0.020	−0.071	−0.100

**Table 2.** Variations of the field of view for the system. Variations No. 1 and 5 are selected along the y directions, No. 3 along the x direction, and No. 2 and 5 along the diagonal directions.

***No.***	**1**	**2**	**3**	**4**	**5**
*θ_x_*(°)	0.000	0.012	0.017	0.012	0.000
*θ_y_*(°)	0.017	0.012	0.000	−0.012	−0.017
